# Multiple root canals in the maxillary molar: an unusual case report

**DOI:** 10.1186/s12903-021-01771-1

**Published:** 2021-08-30

**Authors:** Yao Lin, Yan Xiang, Xiaoling Chen, He Wang, Na Cao, Xiaoman Xu, Yangan Zhang, Zhaojun Wu

**Affiliations:** 1 Endodontics Department of Stomatological Hospital of Xiamen Medical College, Xiamen, 361008 China; 2 Xiamen Key Laboratory of Stomatological Disease Diagnosis and Treatment, Huli District, No. 1309, Lvling Road, Xiamen, 361008 Fujian China

**Keywords:** Microscope, Root canals, Cone-beam computed tomographic, Mesotaurodonts

## Abstract

**Background:**

The objective of this report was to highlight the importance of using a dental operating microscope (DOM) to locate supernumerary canals and diagnose variations in root canals using cone-beam computed tomographic (CBCT) images.

**Case presentation:**

A 35-year-old Chinese female had repeated swelling in the upper right posterior maxilla for 3 months and was referred to evaluate symptomatic apical periodontitis and mesotaurodonts for upper right first permanent molar and upper right second permanent molar. Root canal therapy was proposed and conducted with the use of DOM and CBCT.

**Conclusions:**

Proper diagnosis and careful clinicoradiological examination are necessary, and it is essential to reinforce the knowledge of the rare morphology of root canals for clinicians.

## Background

A comprehensive understanding of root canal anatomy and the morphology of human dentition is essential for clinicians and a prerequisite for conventional endodontic procedures. Untreated canals or canals that are not entirely sealed often fail endodontic treatment [1]. A taurodont is a rare anatomic variant (0.61%) and aberration in tooth morphology characterized by extension of pulp chambers, apical displacement of pulp floors, and short roots [2]. Indeed, only few case reports exist in the dental literature involving root canal anatomy of the upper molars. These reports are summarized in Table 1, which collectively document a maxillary first molar with three roots and six or more root canals. Of note, there are no cases involving three roots with seven or more root canals in the maxillary second molar.

**Table 1:** Previous report cases with 6 or more root canals in maxillary molars

Root configuration	No. of canals	Root canal anatomy			Authors	Year
		MB	DB	P		
*First maxillary molar*						
3 roots	6	3	2	1	Martínez-Berná et al. [3]	1983
3 roots	6	3	2	2	Bond et al. [4]	1988
3 roots	6	2	1	3	Maggiore et al. [5]	2002
4 roots (MB, MP, P, DB)	6	MB, MP, M, P, DP, DB			Adanir [6]	2007
3 roots	6	2	2	2	de Almeida-Gomes et al. [1]	2009
3 roots	6	2	2	2	Albuquerque et al. (3 cases) [7]	2010
3 roots	6	2	2	2	Karthikeyan et al. (4 cases) [8]	2010
3 roots	7	3	2	2	Kottor et al. [9]	2010
3 roots	8	3	3	2	Kottor et al. [10]	2011
3 roots	6	3	2	1	Sharath Chandra et al. [11]	2012
3 roots	7	3	2	2	Gautam et al. [12]	2014
3 roots	6	2	2	2	Present cases	2015
*Second maxillary molar*						
3 roots	6	2	1	3	Pasternak et al. [13]	2007
3 roots	7	3	2	2	Present cases	2016

The present clinical report describes a rare maxillary first molar with six root canals and an ipsilateral maxillary second molar with seven root canals, both of which were shown to be taurodontism. Incorporating a dental operating microscope (DOM) and cone-beam computed tomographic (CBCT) imaging into clinical endodontic practice will increase clinician knowledge and awareness of anatomic complexities. Successful dredging of all root canals in this patient was facilitated with a DOM and confirmed with CBCT images.

## Case presentation

**Fig. 1:**
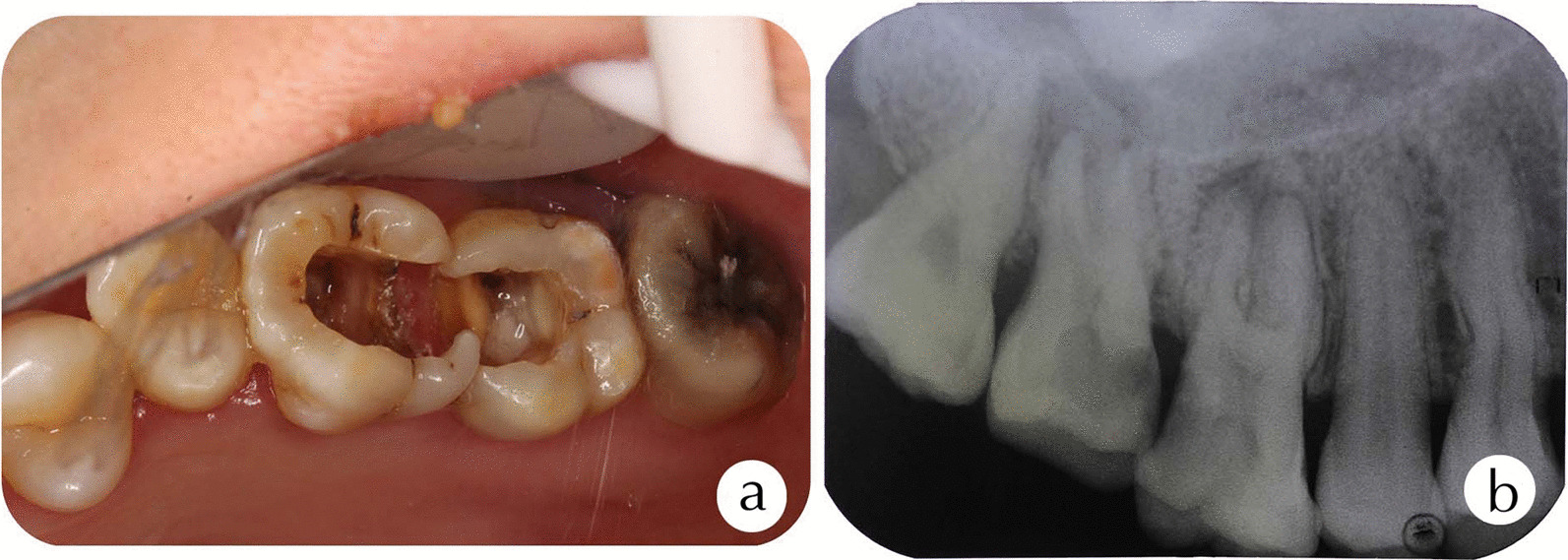
**a** The intraoral examination of teeth #16 and #17. **b** A preoperative radiograph of teeth #16 and #17

A 35-year-old Chinese female was referred to complete root canal treatment of the maxillary right molars using a DOM. She had repeated episodes of swelling in the upper right posterior maxilla for the past three months. The basis for the referral was the presence of a second root canal in the mesiobuccal root (MB2), as stated by the general dentist was suspected but not found. The pulp of 3 main root canals for the upper right first permanent molar (#16, Fédération Dentaire Internationale notation) and upper right second permanent molar (#17, Fédération Dentaire Internationale notation) were removed by the dentist. The medical history was non-contributory. The intraoral examination revealed profound disto-occlusal destruction in #16 and mesio-occlusal destruction in #17 (Fig. 1a). Teeth #16 and #17 were sensitive to palpation and percussion but were negative for thermal testing.

Based on the preoperative radiographs of teeth #16 and #17, the pulp chamber was noted to be extended, the pulp floors exhibited apical displacement, and the roots were short. In addition, teeth #16 and #17 had periapical translucency (Fig. 1b).

According to the radiographic images, teeth #16 and #17 were diagnosed as symptomatic apical periodontitis and mesotaurodonts.

The clinical condition was explained to the patient, and root canal therapy was proposed and conducted. After proper disinfection and rubber dam isolation, all the subsequent procedures were performed using a DOM (Leica, Germany). The residual decay was excavated, and the access opening was prepared. The pulp chamber was identified with three orifice openings (MB, DB, and P), and the dentist utilized the ProTaper F2 after discussion. A complex pattern of the dentinal map in teeth #16 and #17 was demonstrated. After removing the dentinal lips around the orifice of the three prepared canals with a DG-16 endodontic explorer, the second canals (MB2, DB2, and P2) of the tooth #16 were identified. In contrast, the extra root canal orifice was concealed approximately 2–3 mm under the prepared canal orifice of MB and P in the tooth #17.

Two experienced operators (He Wang and Na Cao) analyzed the data taken by CBCT using a Scanora ® 3D unit (SoredexOy, Tuusula, Finland). In this device, the mandible is stabilized with a cheek rest while the patient is seated, and two vertical plastic rods (one on each side) are used to support the head position. The settings (FOV and voxel resolution) were chosen for each patient based on the area to be examined and the diagnostic task in question. Considering the small FOV (6 × 6 cm, resolution 0.13-mm), the scan time was 23 s.

**Fig. 2:**
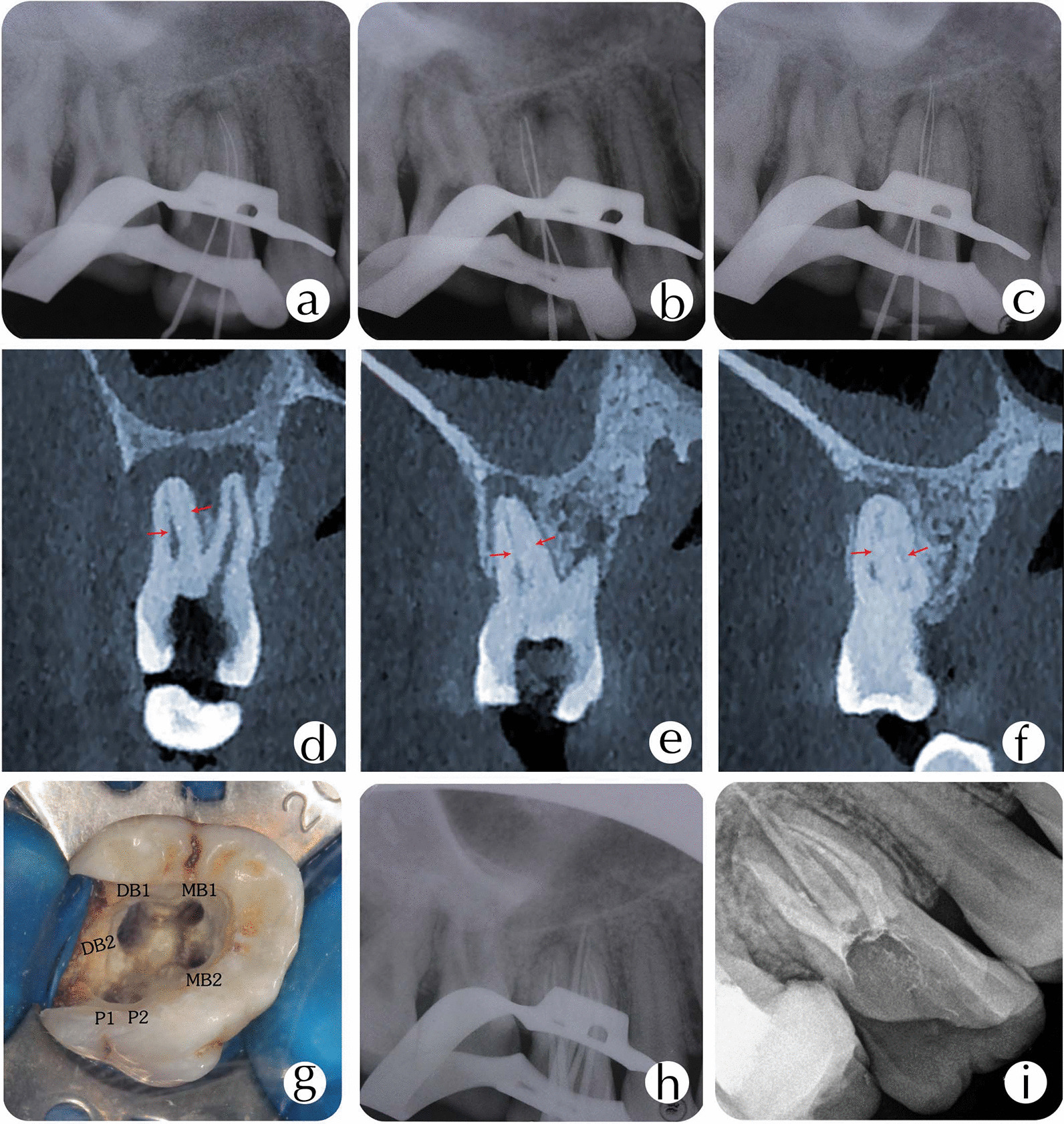
Working length radiograph of tooth #16 in the **a** mesiobuccal, **b** distobuccal, and **c** palatal roots. The MPR views of CBCT showing canal configuration in the **d** mesiobuccal root canal, **e** distobuccal root canal, and **f** palatal root canal. **g** Access opening showing the six root canal orifices. **h** Master cone fit. **i** Post-obturation radiograph

**Fig. 3:**
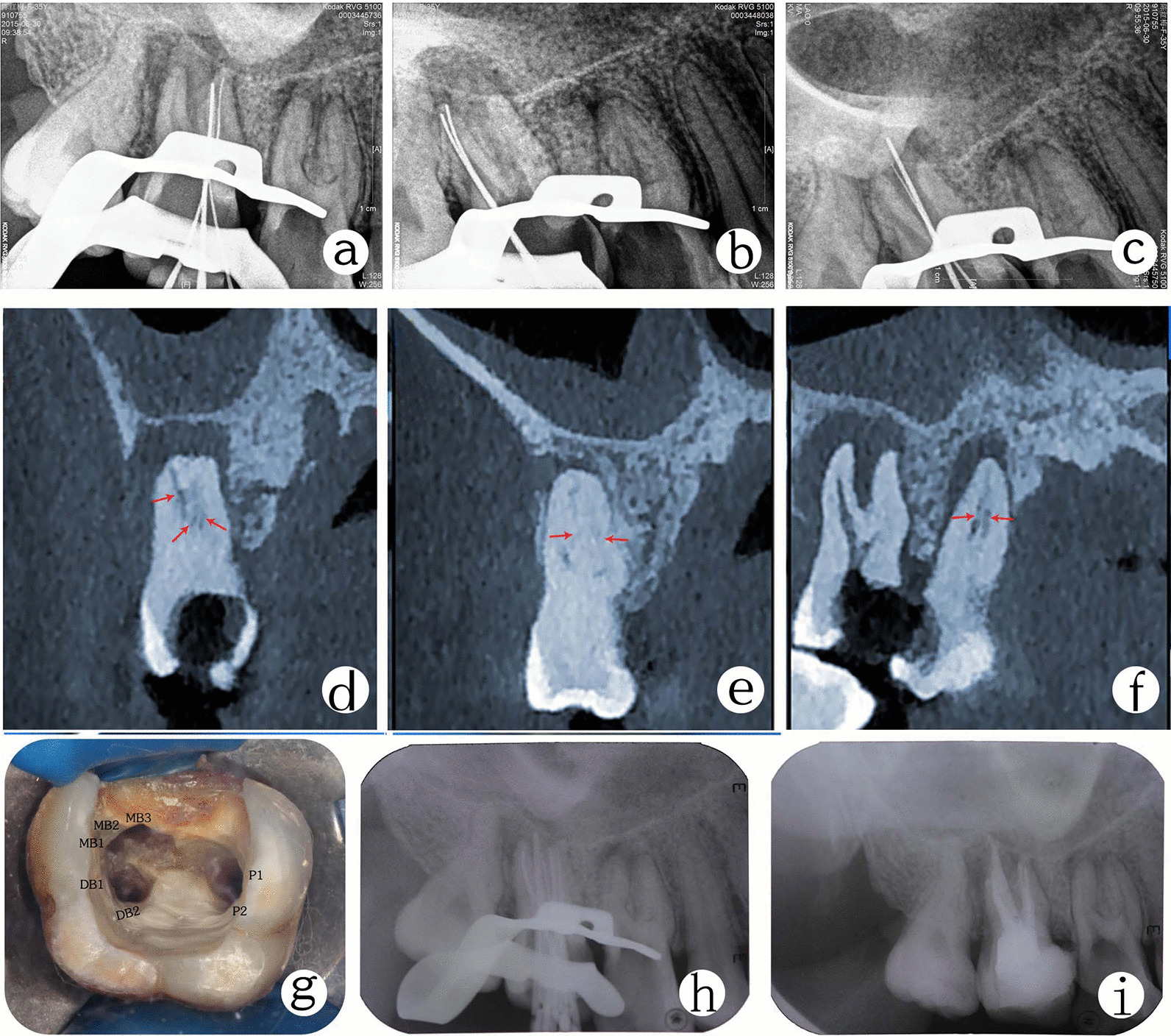
Working length radiograph of tooth #17 in the **a** mesiobuccal, **b** distobuccal and **c** palatal roots. The MPR views of CBCT showing canal configuration in the **d** mesiobuccal root canal, **e** distobuccal root canal, and **f** palatal root canal. **g** Root canals orifices. **h** Master cone fit. **i** Post-obturation radiograph

**Fig. 4:**
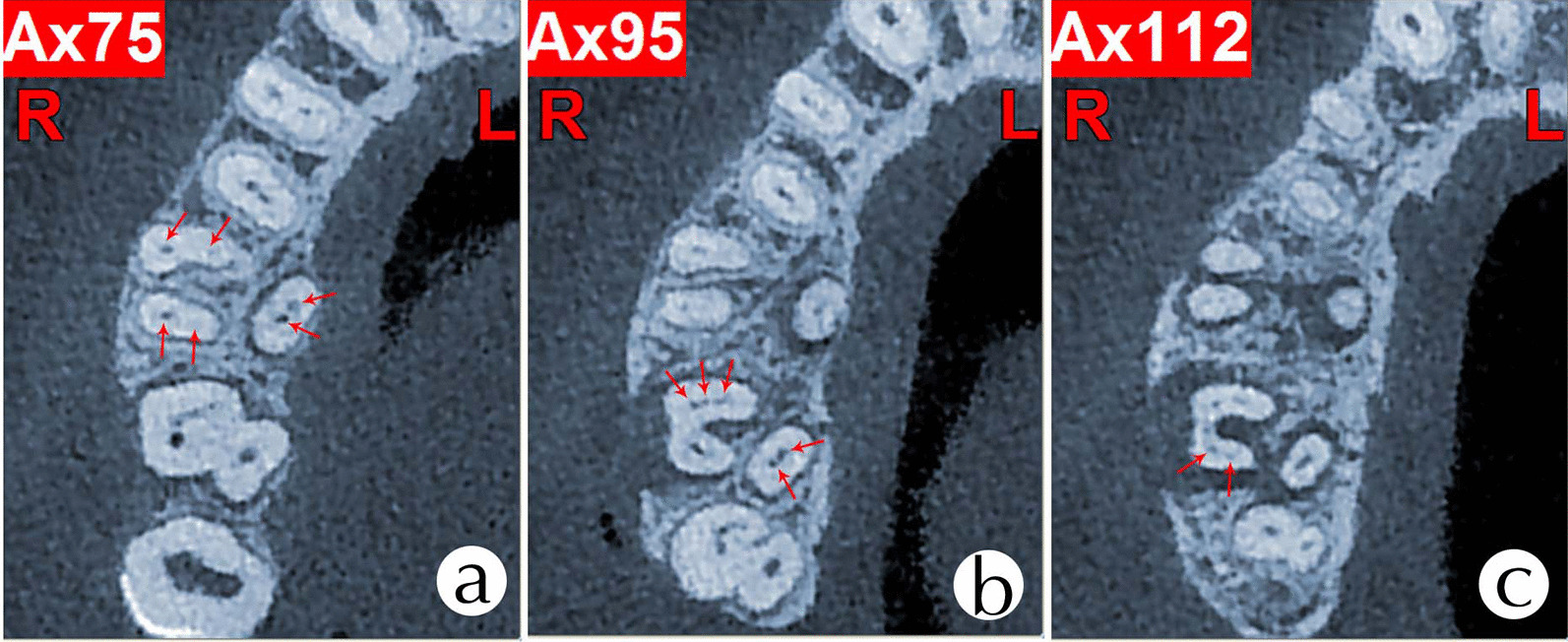
CBCT axial slice showing the **a** coronal, **b** middle, and **c** apical thirds of teeth #16 and #17: Arrow showing the **a** six root canals of tooth#16 and three mesiobuccal root canals. **b** Two palatal root canals of tooth #17. **c** Two distobuccal root canals of tooth #17

The results were further evaluated and verified by the CBCT (Figs. 2, 3, 4). Together with the DOM, the ultrasonic tips maintained good visibility of the operative field. The ultrasonic tips were used for the removal of gross tissue and calculus. We prepared six canals in the tooth #16 with two canals in the mesiobuccl root (MB1 and MB2 canals), two in the distobuccal root (DB1 and DB2 canals), and two in the palatal root (P1 and P2 canals) (Fig. 2a–f). Also, we prepared seven canals in the tooth #17 with three in the mesiobuccal root (MB1, MB2, and MB3 canals), two in the distobuccal root (DB1 and DB2 canals), and two in the palatal root (P1 and P2 canals) (Fig. 3a–f). All of the extra canals were prepared using a stainless steel hand files (ISO size 8). During the root canal preparation, it was apparent that MB2 and MB3 in the tooth #17 were joined together in the apical third of the mesiobuccal root, but P1 and P2 in the same molar were separated in the middle third of the palatal root and joined together in the third apical. The working length was determined by both radiographs and an electronic apex locator (Raypex5; VDW, Germany). The instrumentation was completed using ProTaper sequence S1, S2, F1, and F2 rotary files (ProTaper Universal; Dentsply Maillefer, Switzerland) as per instructions from the manufacturer. Sodium hypochlorite (2.5%) was used as intracanal irrigat, and calcium hydroxide was used as a disinfectant. The access cavity was sealed with a temporary filling (IRM; Dentsply).

One week later, the two teeth were not associated with symptoms, and root canals were obturated using a continuous-wave condensation technique with thermoplasticized gutta-percha (E&Q Plus system; Meta, Korea) and AH Plus (Dentsply) as sealer cement (Figs. 2g–i, 3g–i). The access cavity was temporarily restored, and the patient was sent for coronal rehabilitation.

## Discussion and conclusion

The case was a challenge because of the large morphologic variations of the deep pulp chamber, posterior location, and poor vision. The calcifications covered the extra canal orifice in type II and IV configurations, and the apically dividing systems in type V, VI, and VII could not be detected [3]. The main canals require significant preparation. In the past, we generally accepted that the most common form of maxillary molars had three roots and three canals. In the Hess and Zurcher 1925 landmark study [4], the upper molar mesiobuccal root existed in MB2. Domestic and foreign scholars have studied the existence of MB2. The reported incidence of MB2 in the mesiobuccal root is 51.5–96% based on in vitro results, 16–78% based on in vivo results in the maxillary first molar [5, 6] and 35–94% based on in vitro results, and 9.6–38% based on in vivo results in the maxillary second molar [7, 8]. Zheng et al. [9] indicated that there was a low incidence (0.31%) of 775 CBCT images of maxillary first molars with six root canals; however, anatomic variation studies in the second maxillary molars were not numerous. Pasternak et al. [10] described a second maxillary molar with six canals.

The etiology of taurodontism is still unclear. Most scholars believe that the etiology of taurodontism might be a failure of invagination of the Hertwig epithelial root sheath at a proper spatial plane [2, 11, 12]. Taurodontism is linked to genetic or developmental disorders [13] and is also related to independent disease [2]; The diagnosis of taurodontism is based on the taurodontic index (TI), which is obtained by dividing the height of the pulp chamber by the distance between the occlusal end of the pulp chamber and the root apex [11]. If the TI is ≥ 0.2 mm and the distance between the roof of the pulp chamber and cementoenamel junction (CEJ) exceeds 2.5 mm, then it is defined as taurodontism [11]. Taurodontism may be classified as hypotaurodonts (TI: 0.2–0.3 mm), mesotaurodonts (TI: 0.3–0.55 mm), and hypertaurodonts (TI: 0.55–0.8 mm) according to the severity of taurodontism [12]. Pulp therapy on taurodontic teeth is challenging [14]. Ronald et al. [15] found that taurodontic teeth may have a stunted deformity and tapered root canal. We presented herein a difficult root canal treatment; thus, more care and patience are necessary.

CBCT is an essential diagnostic tool in endodontics for analyzing root canal anatomy and morphology of human dentition, and it gives images which are visualized from multiple orientations in very thin slices without disturbing the overlapping structures [9]. CBCT can provide images at a lower radiation dose with sufficient spatial resolution for endodontic diagnosis and treatment planning [16]. CBCT data have been instrumental in assessing the root and canal morphology in the present case. From the cross sections of the CBCT, a long-narrow mesiobuccal root canal and a rounded or ovoid distobuccal root canal in the maxillary first molar was observed. We also observed that the mesiobuccal and distobuccal root canals in the maxillary second molar were long and narrow, and had a C-shaped buccal root. The multiplanar reconstruction (MPR) of CBCT images showed that the mesiobuccal root canal contained a Vertucci type IV canal, and the distobuccal root contained a Vertucci type IV canal. In contrast, the palatal root showed a Vertucci type II canal configuration in the maxillary first molar. The mesiobuccal root contained a Sert and Bayirli type XV canal, and the distobuccal root contained a Vertucci type V canal. In contrast, the palatal root showed a Vertucci type III canal configuration in the maxillary second molar.

With routine use of the DOM, specific instruments are necessary to increase the clinical procedure's efficiency and effectiveness. The higher magnification and illumination can be used for locating supernumerary canals and improving the discovery of root canal orifices [17]. Studies have shown that the combined use of magnification and CBCT images significantly facilitates the location and negotiation of root canals in upper molars [16, 18].

A number of canals and morphologically abnormal canals can exist in a tooth with vital pulp; thus, proper diagnosis and careful clinicoradiological examination are required. We demonstrated the possibility for complex maxillary molar variations and the need to reinforce clinician knowledge of the rare morphology of root canals.

## Data Availability

The datasets used or analyzed during the current study are available from the corresponding author upon reasonable request.

## Abbreviations

DOM: Dental operating microscope
CBCT: Canals using cone-beam computed tomographic
TI: Taurodontic index
MB: Mesiobuccal root
MPR: Multiplanar reconstruction
